# Heat and carbon uptake in the Southern Ocean: the state of the art and future priorities

**DOI:** 10.1098/rsta.2022.0071

**Published:** 2023-06-26

**Authors:** Andrew J. S. Meijers, Corinne Le Quéré, Pedro M. S. Monteiro, Jean-Baptiste Sallée

**Affiliations:** ^1^ British Antarctic Survey, Cambridge, UK; ^2^ University of East Anglia, Norwich, UK; ^3^ Stellenbosch University, Stellenbosch, Western Cape, South Africa; ^4^ Sorbonne Universite, Paris, Île-de-France, France

**Keywords:** oceanography, Southern Ocean, ocean carbon

The Southern Ocean is an extreme environment. The vast area it covers, roaring winds, mountainous seas and treacherous ice all combine to make it both a challenge and a privilege to study. While researchers no longer take their lives in their hands to travel to the Southern Ocean, as scientists and explorers did in earlier times, it still exerts an undeniable draw on us. It is perhaps fortunate that this draw does exist; research over the last several decades has steadily revealed that the Southern Ocean has an impact on our global climate far exceeding its area and belying its remote nature.

Much, or perhaps even most, of this impact may be attributed to the unique nature of the ocean circulation around the Antarctic, most clearly characterized by the mighty Antarctic circumpolar current (ACC). However, while this horizontal current is the strongest on Earth and forms a critical barrier to the transport from the subtropics to the Antarctic continent, it is the vertical circulation that makes the Southern Ocean truly central to the global climate story. The unique combination of the unblocked circumpolar flow of the ACC, strong meridional density gradients and powerful prevailing winds all act in concert to drive strong vertical transports of water masses. These act to upwell old circumpolar deep water (CDW) on the southern side of the ACC, exposing water to the atmosphere for the first time in several centuries and outgassing some of the natural carbon stored in the deep ocean back to the atmosphere. Driven by easterly winds and intense winter cooling and sea ice brine rejection, a portion of these newly upwelled waters are swiftly subducted again in select regions around the Antarctic coast as Antarctic bottom waters (AABW). These flow north again, filling the global abyss and renewing the very deepest layers as far away as the North Pacific. The other fraction of upwelled CDW is transformed and lightened by melting sea ice and advected northwards by the prevailing westerlies. During this transit, it takes up heat from the atmosphere and absorbs anthropogenic carbon dioxide from the atmosphere. On the northern side of the ACC, a combination of wind and winter heat loss act to subduct these waters as newly formed SubAntarctic mode (SAMW) and Antarctic intermediate waters (AAIW). These are then exported below the pycnocline and into the subtropics, sequestering their contents for decadal to centennial timescales. Ecosystems respond to the transport of nutrients by ocean currents and to regional environmental conditions to modulate the uptake and release of carbon to the atmosphere throughout the Southern Ocean.

Intense research over the past decades has revealed the above storyline to us, and the impact of this vertical circulation on the global climate; the uptake of three quarters of all anthropogenic heat and a quarter of all anthropogenic carbon has become almost canonical in the literature (and especially grant applications). However, many of the processes that contribute to these critical numbers, or even basic characteristics such as the strength, location and variability of these overturning pathways, remain hidden from us. Crucially, the response of future Southern Ocean heat and carbon uptake to projected global warming scenarios, and the nature of the feedbacks that these may generate, are poorly understood and even more poorly constrained by observations.

That is not to say though that researchers have been lax in their duties. Even through the years of restricted field and laboratory work imposed by the global COVID pandemic, progress has steadily been made by the scientific community. In particular, rapid advances and many new questions have been raised through the steady maturation of autonomous observing platforms. These, alongside improved remote sensing and higher resolution models, have brought a much greater appreciation of the scales of variability at play in the Southern Ocean, and the roles they may play in long-term trends.

This special issue arose out of a Royal Society Discussion Meeting, held at the Royal Society in London on 9–10 May 2022, entitled: ‘Heat and carbon uptake in the Southern Ocean: the state of the art and future priorities'. This meeting served as an opportunity for the Southern Ocean heat and carbon research community to come together in person for the first time in several years and appraise the recent progress of the field. It brought together researchers from the fields of physical, carbon/biogeochemical and ecosystem oceanography, covering *in situ* observations, regional and global modelling, and exposing a range of new theory, methods and measurement technologies. The papers arising from this meeting provide a snapshot of the state of the art in Southern Ocean heat and carbon research and serve to highlight advances in understanding over the last several years, as well as identifying new avenues and priorities for future research.

The increasing recognition of the importance of the Southern Ocean in global climate has resulted in it being the focus of large research programmes in recent years, and several of these were highlighted in the Discussion Meeting. The US led Southern Ocean Carbon and Climate Observations and Modelling (SOCCOM) project received much attention, in particular the significant advances in our ability to observe the biogeochemistry of the Southern Ocean that has been delivered by their autonomous float development and deployment. These represent the US contribution to the international Bio-Argo program, the next major step forward for the Argo programme, itself responsible for a major revolution in the way we study and understand the Southern Ocean. With over 270 floats having been deployed at the time of writing, and tens of thousands of profiles now publicly available, these instruments are opening up our understanding of the biogeochemistry of the Southern Ocean, and particularly the carbon cycle. Although, as was discussed at the meeting, these data are not without their difficulties or controversies. In particular, translating *in situ* measurements of pH from these floats to equivalent pCO_2_ for surface flux estimates continues to be a challenge.

Three other large programmes were discussed in the meeting, all focusing on the role of the Southern Ocean in heat and carbon uptake. The first of these was the UK Ocean Regulation of Climate by Heat and Carbon Sequestration and Transports (ORCHESTRA) and its extension; ENCORE is the National Capability ORCHESTRA Extension (ENCORE). This, and the EU funded Southern Ocean Carbon and Heat Impact on Climate, both have made concerted observational efforts to improve process understanding and our ability to model the Southern Ocean, as well as providing wider global contextualization. These presented important findings relating to decreasing trends in Weddell Sea bottom water due to wind changes and the role of the Maud Rise Polynya in regional convection, among numerous other findings discussed respectively in Meijers *et al*. [1] and Sallee *et al*. [2]. Results from the UK Role of the Southern Ocean in the Earth System (RoSES) programme were presented, showing insights into the variability and trends in the Southern Ocean sink for CO_2_ and their driving processes. Similarly, the impact of the South African SOCCO programme was also discussed in several talks, demonstrating advances in our understanding of the role of storms in driving surface CO_2_ and heat fluxes, and influencing the biological carbon pump.

A theme that emerged consistently throughout the Discussion Meeting was the importance, and present poor state of observational coverage enabling estimations of air-sea fluxes. In particular, the lack of wintertime observations of air-sea heat fluxes is especially stark. There is only one ongoing timeseries from the Southern Ocean at present (the SOFS mooring just south of Tasmania), with the complimentary OOI mooring in the southeast Pacific having recently been removed. The need for more such moorings, particularly in the presence of zonal variations in air-sea heat flux, is presented in Josey *et al*. [3]. Likewise, wintertime observations of surface ocean pCO_2_ are also sparse, although they have been growing with the recent SOCCOM and other ARGO programmes. By contrast, there has been an explosion of new high-resolution observations of the Southern Ocean mixed layer facilitated by the rapid proliferation of autonomous observation platforms, particularly subsurface gliders and surface vehicles. Swart *et al*. [4] describe recent advances in characterizing and understanding the fine scale structure of the surface Southern Ocean revealed by such instrumentation, as well as discussing the challenges inherent in reconciling such high frequency data with more traditional views of the ocean from hydrography and reanalysis datasets.

Bringing these themes together, Tamsitt [5] summarizes the recent advances in our understanding of mixed layer variability in the region of SubAntarctic mode water (SAMW) formation, the critical regions of subduction in the upper overturning cell. The maturation of the Argo dataset to (almost) multi-decadal length has revealed significant spatial and interannual variability in the Pacific and Indian SAMW pools, and the heat flux moorings have been instrumental in demonstrating the importance of regional meridional wind anomalies in setting this variability.

The role of sea ice melt in both setting water mass transformation rates and driving trends in the Southern Ocean has become clearer in recent years. The emergence of this view coincides with record variability in Antarctic sea-ice area, and perhaps even a shift towards a reducing trend after several decades of quasi-stability. Meredith *et al.* [6] demonstrate the emerging utility of isotopic techniques in ascribing sources to freshwater anomalies and can distinguish between meteoric and sea-ice sources. They apply this technique using oxygen 18 to 16 ratios to demonstrate the freshening of the northern Weddell Sea in response to anomalous sea ice losses in 2015/16.

The latter half of the meeting turned to the critical issue of carbon flux, storage and transport in the Southern Ocean. This has been the subject of intense study, as the Southern Ocean at the latitudes of the ACC is both a significant source to the atmosphere of natural CO_2_, as well as a major global sink of anthropogenic carbon. This concerted effort has led to the development of important community databases (notably SOCAT, the Southern Ocean CO_2_ Atlas), but there are significant seasonal biases in these ship-based data, and very little pCO_2_ data exists south of the Polar Front during austral winter. Landschutzer *et al*. [7] document how a new type of dataset coming from sailboats, particularly racing craft, navigating the Southern Ocean can help fill the observational gap and be useful in our understanding of Southern Ocean carbon budget.

The emerging complexity of the biological component of the Southern Ocean carbon cycle is addressed in Ahugerahanti & Tagliabue [8] who revisit the paradigm of micronutrient limited primary productivity and explore the processes controlling iron and manganese co-limitation. As in the physical and biogeochemical circulation systems, autonomous instrumentation has provided new insights into the fine scale structures of the biological carbon pump, and Thomalla *et al*. [9] explore what the new resolution of seasonal timescales can reveal about the impact of climate change on this system.

Finally, the discussion turns to the future of the Southern Ocean, and the impacts that climate change will have on its ability to continue to sequester and export heat and carbon. Hauck *et al*. [10] describe the difficulties in accurately assessing past trends in the global ocean carbon sink, and particularly the importance of biases induced by data sparsity in the Southern Ocean, which they suggest lead to significant overestimates of the strength of the carbon sink. Mayot *et al*. [11] demonstrate that presently process based global ocean models including carbon cycles and forced by reanalysis atmospheres cannot recreate the observed interdecadal variability inferred from observational CO_2_ flux products. They also show that while there are questions around the validity of these observed CO_2_ signals, due to data sparsity, they are at least broadly consistent with independent O_2_ flux products. They hypothesize that the model-observation differences may be due to a poor representation of the thermal/non-thermal components of the Southern Ocean sink, or ongoing problems in resolving mode water formation in low resolution models.

To bring a global perspective, Williams *et al*. [12] examine the role of the Southern Ocean in setting global ocean heat uptake in coupled climate models, as well as its impacts on climate metrics such as the transient climate response where it exerts an impact greater than its proportional area. They find that when examining simulations forced with CO_2_, as opposed to a full anthropogenic and natural suite of variability, the Southern Ocean's role in heat uptake is significantly reduced, while its importance for carbon remains, a result potentially due to uneven thermal forcing in ‘historical' scenarios by aerosols.

These papers provide a valuable overview of the status of present research into the Southern Ocean's critical role in carbon and heat uptake, storage and export. Significant advances have been made, with notable discoveries of regional interannual variability, an increased appreciation for the role of zonal variability in setting integrated circumpolar properties and the emergence of fine structures at high temporal frequencies as a dynamic new field of study. However, the field still struggles to estimate the decadal variability, or even mean trends, in the overturning circulation or resolve those factors driving its variability. Additionally, we remain far from fully characterizing the biological components of the carbon cycle and have critical gaps in our observation of surface fluxes of all forms. These papers demonstrate great progress, and especially the role of new technologies in driving forward our understanding, but there is still much work to be done in understanding this vital ocean at the end of the Earth.

## Data Availability

This article has no additional data.
